# Primary care patients’ experiences of video consultations for depression and anxiety: a qualitative interview study embedded in a randomized feasibility trial

**DOI:** 10.1186/s12913-022-09012-z

**Published:** 2023-01-04

**Authors:** Markus W. Haun, Lydia Oeljeklaus, Mariell Hoffmann, Justus Tönnies, Michel Wensing, Joachim Szecsenyi, Frank Peters-Klimm, Regina Krisam, Dorothea Kronsteiner, Mechthild Hartmann, Hans-Christoph Friederich

**Affiliations:** 1 Department of General Internal Medicine and Psychosomatics, Heidelberg University, Im Neuenheimer Feld 410, D-69120 Heidelberg, Germany; 2 Department of General Practice and Health Services Research, Heidelberg University, Heidelberg, Germany; 3 Institute of Medical Biometry and Informatics, Heidelberg University, Heidelberg, Germany

**Keywords:** Primary health care, Telemedicine, Remote consultations, Depression, Anxiety, Qualitative research, Mental health services

## Abstract

**Background:**

Integrated mental health care models that provide rapid access to video consultations with mental health specialists for primary care patients are a promising short-term, low-threshold treatment option and may reduce waiting times for specialist care. This qualitative study, nested within a randomized feasibility trial, aimed to explore participants’ views on this type of care model, its influence on the lived experience of patients, and barriers and facilitators for its delivery.

**Methods:**

In five primary care practices, 50 adults with depression and/or anxiety were randomly assigned to either an integrated care model (maximum of five video consultations with a mental health specialist) or usual care (primary care or another treatment option). Prior to obtaining the trial results, interviews were held with participants who had received video consultations. Interviews were transcribed and analysed thematically.

**Results:**

Twenty of the 23 patients who received video consultations participated in the interviews. Patients engaged well with the care model and reported positive effects on their most pressing needs, while denying safety concerns. Generally, they perceived the usability of video consultations as high, and temporary connectivity failures were not considered a substantial barrier. We identified two key mechanisms of impacts on the patients’ lived experience: fast access to specialist mental healthcare and the emerging rapport with the specialist. In particular, patients with no prior mental healthcare experience indicated that familiarity with the primary practice and their physician as a gatekeeper were important facilitators of proactive treatment.

**Conclusions:**

From the patients’ perspective, mental health care models integrating video consultations with mental health specialists into primary care are linked to positive lived experiences. Our findings imply that primary care physicians should promote their role as gatekeepers to (1) actively engage patients, (2) apply integrated care models to provide a familiar and safe environment for conducting mental health care video consultations, and (3) be able to regularly assess whether certain patients need in-person services. Scaling up such models may be worthwhile in real-world service settings, where primary care physicians are faced with high workloads and limited specialist services.

**Trial registration:**

DRKS00015812.

**Supplementary Information:**

The online version contains supplementary material available at 10.1186/s12913-022-09012-z.

## Background

### Primary mental health care

Depression and anxiety are among the most disabling mental health conditions worldwide [1–3] and are often managed entirely within primary care [4–6]. Primary care physicians (PCPs) provide comprehensive care to most of their patients, who in turn mostly prefer to be cared for by their PCP [7, 8]. However, PCPs already struggling with high workloads increasingly see patients suffering from physical-mental multimorbidity and/or immobility in aging societies [9–12]. Moreover, given the rising number of patients with an urgent need for support, PCPs and patients are confronted with long waiting lists for specialist mental health care [13].

### Technology-based integrated mental health care

Given these challenges, integrated care approaches that embed mental health specialists (MHS) directly in primary care are increasingly implemented [14–19]. These approaches propose features such as multidisciplinary teams working collaboratively with each other and patients in the same location [14], telepsychiatry-enhanced integrated care (e.g., for direct evaluation of patients at an originating site by a psychiatrist at a distant site for difficult-to-reach areas) [15], or unified patient care plans addressing both mental health issues and health behavior changes (e.g., diet or avoidance of recreational drug use) [16]. Recently, with the aim of improving compatibility with small and/or remote primary care practices, integrated care has been expanded by introducing video-based integrated mental health care [20–27]. Leveraging expertise at a distance, MHS located off-site provide video consultations to patients presenting with mental health conditions in primary care practices [28]. PCPs and their patients have increasingly used video consultations as an alternative to face-to-face consultations in recent years. Depending on the context, physicians see the benefits in this mode of delivery [29]. For appointments that do not require physical examination or when the patient is immobile, video consultations are efficient, and physicians do not feel that their work routine is impaired [30]. Patients realize the advantages in terms of convenience and access to mental health care as video consultations can reduce travel costs and time [31]. Many report feeling able to establish rapport with their physicians. While guidance for conducting remote consultations is available [32–34], evidence of the effectiveness and implementation of a transdiagnostic approach for managing mental health conditions through video-based integrated mental healthcare is scarce.

Involving key stakeholders, the PROVIDE (ImPROving cross-sectoral collaboration between primary and psychosocial care: An implementation study on VIDEo consultations) project developed such a model featuring mental health specialist video consultations for patients in primary care [35–37]. The project progressed with a randomized pilot trial (PROVIDE-B) evaluating the feasibility of mental health specialist video consultations in 50 patients with depression and/or anxiety disorders presenting to German primary care practices [38, 39]. In Germany, PCPs are reimbursed through regionally negotiated fee-for-service payments up to the maximum number of services per quarter. Generally, there is no gatekeeping and patient registration is not required (free-access system), but health insurances are required to offer the option to enroll in a family physician model with gatekeeping [40]. Remote consultations are not regularly provided by German PCPs, although video consultations are covered by all health insurances.

In PROVIDE-B, patients were randomized into two groups receiving either treatment as usual, as provided by their PCP, or up to five video consultations conducted by an MHS. The video consultations focused on (1) systematic diagnosis and proactive monitoring using validated clinical rating scales, (2) the establishment of an effective working alliance, and (3) a stepped-care algorithm within integrated care-adjusting treatments based on clinical outcomes. The trial yielded a high consent and retention rate [39]. In this paper, we report our qualitative findings.

### Purpose of the study

Giving voice to patients suffering from depression and/or anxiety, this qualitative process evaluation nested within the PROVIDE-B randomized pilot study aims to investigate the implementation of the video-based integrated mental health care model. Specifically, we investigate patients’ perspectives on (a) how well the mental health specialist video consultations were delivered and received (implementation), (b) how the mental health specialist video consultations affected the patients’ mental health (mechanism of impact), and (c) which external factors influenced the delivery of consultations (context).

## Methods

### Study design

We conducted semi-structured interviews with patients participating in the assessor-blinded, randomized, prospective, parallel group PROVIDE-B trial, which aimed to assess the feasibility of the intervention and study procedures and was therefore not sufficiently powered to detect treatment differences between the intervention and the control group [41–43]. We took a critical realist stance to conduct the study, that is, while assuming social structures independent of our understanding (e.g., fear of stigma), we examined how participants constructed meanings when engaging with these structures (e.g., what does a fear of stigma mean for the respective individual?) and in doing so aimed to account for our own experience and background as researchers (e.g., how clinicians contribute to stigma) [44].

### Study setting

PROVIDE-B recruited 50 participants with depression and/or anxiety from five primary care practices in the State of Baden-Wuerttemberg in Southern Germany [38]. The practices were typical of German primary care with respect to the age of the PCP (mean = 58.5, standard deviation = 50.8); size of the practice (average number of patients per quarter: two practices with 501–1000 patients, one practice with 1001–1500 patients, two practices with > 1500 patients); and the degree of urbanization of the area the practices were located in. The care model featured a targeted primary care-based mental health intervention that combined elements of the collaborative care and consultation-liaison models [45–47]. As for the mode of delivery, the model featured web-based video consultations through a primary care practice with patients who were in the primary care practice for consultations and mental health specialist at a distant site. We recruited four mental health specialists at the Institute for Psychotherapy, Heidelberg, which is a state-approved psychotherapeutic training facility located at Heidelberg University. The mental health specialists were clinical psychologists with a diploma or master’s degree in psychotherapy training or resident doctor training for board certification in psychosomatic medicine and psychotherapy, which is an independent specialty in Germany. All participating specialists had at least two years of training. Although specialists were not allowed to prescribe medication owing to regulatory restrictions, they could suggest starting the patient on medication or changing their medication.

The patients were recruited via their PCPs during regular visits. Based on their clinical judgment, the PCPs prospectively selected individuals suspected to be affected by depression or anxiety and presented the study to them by offering informational material. To screen them objectively, we then conducted standardized Computer Assisted Telephone Interviews using the Patient Health Questionnaire 9 and the General Anxiety Disorder 7instrument [48] to determine whether they met the inclusion criteria to suffer from an at least moderate severity of depression and/or anxiety. After providing written informed consent, eligible participants were randomly assigned (1:1) to the video consultation group or treatment as usual group via a secure web-based randomization system.

Patients in the intervention group were scheduled at biweekly intervals for up to five sessions lasting 50 min each. The intervention followed a transdiagnostic treatment approach for emotional disorders [49, 50]. Specifically, it included three core intervention elements for effective primary care-based mental health care: (1) systematic diagnosis and proactive monitoring using validated clinical rating scales, (2) establishment of an effective working alliance, and (3) a stepped-care algorithm within integrated care. If indicated, the intervention also included brief problem-solving therapy [51, 52]. The video consultations were conducted on an encrypted web-based videoconferencing platform on a subscription basis (arztkonsultation ak GmbH; https://arztkonsultation.de) at fixed time slots set by the primary care practice staff. Patients engaged in the videoconferences in a designated room in the primary care practice and the mental health specialists used either their office/private practice or another suitable designated room at home. By locating the patients in the primary care practice, we ensured that the technical requirements for conducting video consultations (e.g., stable internet connection, hardware such as webcams) were met and a safe and appropriate environment was provided for the duration of the consultation, which may not be the case for all patients at home. As the video conferencing platform was easy to access, patients who had different levels of experience with videoconferencing had no major difficulties logging in. Each mental health specialist was permanently assigned to one primary care practice so that each patient who received video consultations was seen by the same therapist over the five sessions they were given. Patients allocated to the control group received the usual care provided by the PCP. Details of the trial can be found elsewhere [38].

### Recruitment and sampling

We invited all 23 patients from the intervention group to participate in an interview, three of whom declined, stating that they did not have time for an interview. In total, we interviewed 20 study participants (median: 23 minutes; interquartile range: 9 minutes).

### Data collection

We designed and piloted a semi-structured interview guide (see Additional file 1). The topics of interest were discussed during research team meetings, and MaHo developed the first version of the interview guide. After the review and adjustments, it was used in the first two interviews. We then re-reviewed it to check whether the original version of the interview guide had failed to capture any key aspects expressed by the patients. As this was not the case, this version was used for the rest of the interviews. MaHo (a sociologist/PhD student, > 6 years of experience with qualitative methods) conducted and audio-recorded the telephone interviews without having interacted with the participants before. MaHo discussed the progress of sampling and data collection with the MWH (an MD/psychologist, > 10 years of experience with qualitative methods). We collected the sociodemographic and medical characteristics of all the participants as part of the baseline assessment of the trial. Data were collected by phone between June and December 2019.

### Data analysis

Two coders (MaHo and LO), who were not involved in the delivery of the intervention, analyzed the data prior to knowing the quantitative trial outcomes [43]. The coders independently conducted a thematic analysis of seven transcripts in MAXQDA, a software program designed for computer-assisted qualitative data analysis that facilitates analysts’ interactions with codes and memos [41]. Both analyses were reviewed using MWH to derive a single robust code system that was applied to the remaining transcripts (see Additional file 2). After all data had been coded, for the intervention group data, we collated the inductively generated themes with the three key aspects of implementation quality (implementation, mechanism of impact, and context), functioning as top-down themes [42]. Theme saturation was reached when the analyzed data did not provide any new themes [53]. To support the credibility of our analyses and review data saturation, we conducted member checks with the participants [54]. After the data analysis was completed, participants received an anonymized written summary of the interview findings and evaluated the extent to which these findings reflected their statements. We contacted all participants via telephone to receive their feedback on the summary prior to utilizing it to discuss the results.

## Results

### Sample

Table 1 presents the interviewees’ characteristics. The baseline assessment of the trial yielded no significant differences between the intervention and control groups in terms of age, sex, or severity of depression and anxiety symptoms. For further information, see our publication on quantitative findings [39].

**Table 1:** Characteristics of the interviewees

	***N*** = 20
**Age**	
Mean (SD^a^)	45.3 (16.3)
Median [Min, Max]	48.0 [22.0, 72.0]
**Gender, n (%)**	
Female	13 (65.0%)
Male	7 (35.0%)
**Country of origin, n (%)**	
Germany	18 (90.0%)
Other	2 (10.0%)
**Marital status, n (%)**	
Single	4 (20.0%)
In a partnership	16 (80.0%)
**Education level, n (%)**	
9 years or less	5 (25.0%)
More than 9 years	14 (70.0%)
Missing	1 (5.0%)
**Employment status, n (%)**	
Employed	12 (60.0%)
On sick leave	3 (15.0%)
Retired	3 (15.0%)
Unemployed	1 (5.0%)
Missing	1 (5.0%)
**Level of depressive symptoms (PHQ-9**^b^**)**	
Blank	1 (5.0%)
Mild	3 (15.0%)
Moderate	12 (60.0%)
Severe	4 (20.0%)
**Level of generalized anxiety (GAD-7**^c^**)**	
Blank	1 (5.0%)
Mild	9 (45.0%)
Moderate	8 (40.0%)
Severe	1 (5.0%)
Missing	1 (5.0%)
**Current psychiatric treatment/psychotherapy**	
No	17 (85.0%)
Yes	3 (15.0%)
**Current psychopharmacological treatment**	
No	13 (65.0%)
Yes	7 (35.0%)

^a^*SD* Standard deviation; ^b^*PHQ-9* Patient Health Questionnaire 9; ^c^*GAD-7* Generalized Anxiety Disorder 7

### Implementation: how well were the mental health specialist video consultations delivered and received?

#### Patients’ perceptions of implementation

Most patients described the mental health specialist video consultation model as a good fit for day-to-day operations in primary care practice, reporting that consultation scheduling proceeded smoothly and coming to the practice for the consultations was feasible. During the video consultations, most patients reported temporary interruptions of audio and/or video transmission (e.g., audio delay) at some point, most likely due to connectivity failures. In addition, a few patients reported feeling distracted by these interruptions to the extent that prevented them from engaging with the mental health specialist.

*The only disadvantage was the [Internet] connection from time to time; sometimes, audio transmission did not work quite well. That’s really a big shortcoming when you talk about something intensively and then the [Internet] connection fails or the sound is delayed. (Participant 20).*

All patients generally felt comfortable with the video consultation as the mode of delivering the intervention, which enabled them to discuss with the mental health specialist the full range of their emotional experiences. A notable proportion of patients considered mental health specialist video consultations to be equivalent to in-person visits.

*The mode of delivery did not matter. For me, this was a personal conversation, and it would not have ended any differently if we had been sitting in the same room. (Participant 02).*

A few patients infavored in-person consultations over mental health specialist video consultations and considered those visits to be “more intense” (Participant 10). Indeed, several patients, all residing in suburban areas, missed having in-person interactions.

*You cannot quite cover the whole spectrum of body language (…*) *You cannot have a complete picture of the video consultation. (…) I think that it makes [it] difficult in some moments when it comes to body language, posture, and facial expressions. (Participant 06).*

The patients did not mention any major safety concerns (e.g., data breaches). In fact, almost all patients viewed the consultations as successful, concluding that the intervention had helped improve their health status.

*I can take a lot of this with me. I have two or three sentences — I also told her [the mental health specialist] — I think about it again and again because they were very helpful to me. (Participant 04).*

#### Suggestions for modification

While most patients felt comfortable sharing their emotional problems solely with the mental health specialist, two patients advocated for closer involvement of the PCP; for example, by providing warm hand-offs to the mental health specialist during the first consultation. Some patients stated that they preferred to participate in consultations from home. Patients with prior experience in videoconferencing were more likely to advocate for consultations from home than patients without prior experience.

### Mechanisms of impact: participants’ accounts of how the consultations affected their mental health

Half of the patients reported entering the first consultation with a rather reserved attitude towards mental health specialist video consultations, regarding them as an impersonal mode of delivering treatment. However, after getting to know the mental health specialist and as the consultations progressed, the patients felt increasingly comfortable. Notably, the video modality became less important and the patients’ focus shifted to their relationship with the mental health specialist, and for most patients, this relationship emerged as a key factor in their treatment. Patients appreciated the compassionate conversations with the mental health specialist, whom they regarded as a non-partisan authority providing a professional perspective on patients’ most pressing clinical problems:

*The conversations were particularly helpful. I had questions, I got answers. I got food for thought. I also got another view, so I got everything I actually expected to get from a psychotherapist and specialist in these things, in this area. (Participant 07).*

Furthermore, fast access to specialist mental health care through mental health specialist video consultations was a key factor for improvement. Given their previous struggles with long waiting times for specialists, patients particularly valued mental health specialist video consultations as a low-threshold modality for timely contact with specialists.

*If I had made an appointment with a psychiatrist or psychotherapist, I would have had to wait months. Now [refers to the mental health specialist video consultation] I had quick access to a therapist. (Participant 21).*

### Context: which external factors influence the delivery and functioning of mental health specialist video consultations?

Familiarity with primary care practice emerged as the main facilitator of treatment engagement. Patients perceived coming to a practice as a more familiar, less stigmatizing, and hence less cumbersome way of contacting the mental health specialist compared to seeing them in an office. Familiarity with primary care practice enabled patients to open up more easily to the mental health specialist. Notably, patients from rural and suburban areas particularly linked the familiarity of the primary practice to beneficial consultations. Moreover, patients with no prior experience in turning to specialized mental health services were more likely to relate the familiarity of the primary care practice to their improvement than those who had previously consulted a mental health specialist. Finally, half of all patients, particularly those from rural areas, indicated that mental health specialist video consultations in primary care practice saved travel time to specialists, who are often located in suburban and urban areas.

### Member checking

The participants agreed that the findings adequately reflected their personal experiences with mental health specialist video consultations. Some patients regarded the non-partisan and protected environment of primary care practice as essential, given that it provided an external perspective:

*At any rate, I advocate for the primary care practice setting; I need to step outside to be able to look on everything from outside – this would not work well from home. (Participant 04).*

## Discussion

### Summary

This study found that patients engaged well with the video-based integrated mental healthcare model, describing the positive effects on their most pressing needs while negating safety concerns. The usability of video consultations was generally perceived as high, and temporary connectivity failures were not regarded as a substantial barrier. We identified two key mechanisms of impact: fast access to specialist mental healthcare and emerging rapport with the specialist. Familiarity with primary care practice was seen as an important facilitator for proactive treatment engagement, particularly for patients from less densely populated areas and patients with no prior mental healthcare experience.

### Strengths and limitations

First, the sample size is relatively small. However, we included patients from all general practices that participated in the feasibility trial. This allowed us to report the experiences of patients from different locations and cover the potentially influencing structural differences in one or more practices. Furthermore, our sample, which was comprised of individuals with mild to moderate symptom severity and mostly lacked current mental health treatment (either psychological/psychiatric or psychopharmacological) seems very relevant to an intervention study in primary care in which this patient profile predominates [55, 56].

Second, we relied on participants’ self-reported intentions and practices, which always carries the risk of social desirability that can bias the findings, particularly after the patients had received the intervention. However, we tried to minimize the limitations of self-reports by firmly reassuring the participants of the confidential nature of their participation and encouraging them to express their opinions and thoughts honestly.

Third, the interviews were rather short. Telephone interviews are typically, and on average, shorter than those conducted face-to-face – an observation that usually results from participants speaking for less time. However, we reverted to a viable strategy for reducing this tendency by (1) reducing the number of themes covered compared to what we had planned initially and (2) providing a highly structured interview guide [57]. In addition, telephone interviews allowed us to include participants across a wider geographical scale and to offer greater anonymity given that mental health is a topic of a sensitive nature [58].

Fourth, experiences from the participants were mostly positive and there may be a lack of more critical perspectives in our sample. Our sampling strategy was to include all patients in the intervention group. Thus, we did not distinguish between positive and negative cases. However, of the 23 participants in the intervention group, three patients did not participate in the interviews. It can be assumed that those who were not interested in participating were more likely to have had negative experiences with video consultation and did not want to participate further in the study. To validate the general feasibility and benefit of this treatment model, future studies should consider this aspect.

Finally, as our trial was completed shortly before the COVID-19 pandemic began, our results were unaffected by the changing conditions of health-service delivery created by the pandemic. However, isolation due to social distancing and repeated lockdowns is a tremendous threat to the mental health of many people for the foreseeable future [59, 60]. In this regard, video consultations offer a safe way for patients, even for groups at high risk in the case of COVID-19 infection, to interact with the healthcare system. Indeed, such consultations have indeed been implemented widely. Our findings highlight the additional potential of video consultations for engaging hard-to-reach patient groups that are prone to be affected by the discontinuity of current care. Nevertheless, the availability of contact information, seamless scheduling of appointments, provision of a private, well-lit room with sufficient bandwidth, and a clear follow-up plan remain hallmarks of a good virtual experience [61].

### Comparison with the existing literature

Observational studies indicate that patients generally welcome telehealth primary care video visits as a reasonable alternative to in-person visits [29, 31, 59, 60]. Nevertheless, our findings add new insights to the existing literature. First, there is preliminary evidence of video consultations being particularly suited for mental health problems [35, 62]. However, in an interview pre-implementation study conducted some time before the PROVIDE-B trial started, patients with no prior experience with video consultations expected several limitations from such consultations (i.e., a more fragile therapeutic relationship owing to the lack of face-to-face contact, technical challenges through connectivity failures, and organizational challenges for the primary care practice staff) [35]. In contrast, the current study, which is one of the first to report the qualitative findings of a randomized trial on a telehealth intervention in primary mental health care, showed that effective therapeutic relationships could be established and consultations implemented in the daily routine of primary care practices without any notable disruptions. While some connectivity failures occurred, participants did not consider them a substantial problem. Second, our findings add to the literature on the distinctive role of PCPs as gatekeepers for video-based integrated mental health care. For example, a trial on home-based telehealth problem-solving therapy (PST) for depressed older adults yielded findings on implementability and benefits that are in line with the patient experience described in our study [63]. Nevertheless, for the community population in the PST trial, the consent rate was as low as 10–20%, potentially owing to a lack of motivation and/or denial of depression. Given the importance of interpersonal trust in telehealth delivery, it seems plausible that in our trial, PCPs may have functioned as key gatekeepers or motivators, thus encouraging patients to engage in the intervention [64, 65]. Third, our findings point to specific target populations for video-based integrated mental healthcare in the post-COVID19 era in that mental health specialist video consultations seem particularly suited for patients from rural areas and those with no prior experience in mental healthcare. Specifically, the reduced travel burden emerged as a facilitator for engaging in mental health specialist video consultations, which is in line with results from a large cross-sectional analysis using health insurance data delineating transportation time as a major in-person visit barrier [66]. Surprisingly, patients with no prior experience in mental healthcare seemed to experience less stigma in our trial. We consider this an important result, given that experiencing less stigma has emerged as a facilitator of treatment engagement in previous stepped-care trials [66, 67]. Notably, patients in our study assigned more importance to the one-on-one interaction with the mental health specialist rather than to specific intervention components, which is in line with findings from the CASPER Plus trial evaluating telephone-delivered behavioral activation in older adults with depression recruited in primary care facilities (63). More recently, it was shown that active engagement of patients through the development of a collaborative and empathic relationship is one of the cornerstones of primary care interventions for mental-physical multimorbidity [67]. Our findings highlight the importance of personal relationships in the context of video consultations—a tenet of a mental health specialist video consultation model embedded in primary care—and leverages ongoing or even longstanding patient-PCP relationships.

Our study also identified challenges and drawbacks associated with video-based integrated care, which are of immediate practical importance given the current all-embracing presence of video conferencing. First, some patients missed the ability to develop a good therapeutic relationship in an in-person encounter with the mental health specialist. This observation resonates with findings from the Virtual Outreach Study on joint teleconsultations between patients, PCPs, and a hospital-based specialist, where patients described a sense of alienation arising from the use of technology [68]. It seems plausible that some people will always need in-person services and the close patient–doctor relationships that emerge from them (e.g., via body language) [69, 70]. In the same vein, some professionals have raised legitimate concerns that doctors will get used to socially distanced medicine, move away from seeing patients face-to-face after the COVID-19 pandemic, and eventually know their patients less than before [71].

Second, although patients did not feel substantially disturbed by connectivity failures, poor network coverage and low bandwidth remain significant barriers for the acceptance of video consultations, particularly in some rural and/or remote areas, which must be addressed through timely and exhaustive broadband expansion [62, 72].

Third, data privacy protection is always an essential prerequisite for the implementation of video consultations. Our findings show that practices manage to regularly provide a quiet and confidential place free from interruptions in consultations. However, while the German government regulations require adherence to the EU General Data Protection Regulation from certified operators (e.g., video and audio communication is not recorded or stored on any server), data breaches can never be fully ruled out for video consultations. Finally, while the expectation of personal benefit is a well-known prerequisite for both patients and health providers to participate in large effectiveness trials [73, 74], one qualitative study of a large cluster-RCT [75] confirmed that health professionals are more doubtful about the use of telemedicine compared to patients [29, 76]. Hence, while there may be a reasonable number of patients in need of a certain catchment area, the implementation of videoconferencing services ultimately depends on the willingness of PCPs to refer patients [77]. In this regard, the COVID-19 pandemic has shifted to remote consulting with PCPs, focusing on vulnerable patients, including those with poor mental health [78].

## Conclusions

From the patients’ perspective, video consultations with mental health specialists in primary care are implementable, safe, and related to a positive impact on an individual’s lived experience. Our findings underscore that PCPs should (1) continue to proactively take on their distinctive role as gatekeepers for actively engaging patients, (2) apply such models to mitigate patients’ fear of being stigmatized for those with no prior experience with mental health care, and (3) regularly check whether certain patients need in-person services and if those receiving video consultations are beginning to feel alienated. Given the widespread nature of the COVID-19 pandemic and the continuing need for social distancing, mental health specialist video consultations embedded in primary care offer a promising approach for addressing pandemic-related decline in mental health and securing treatment engagement in vulnerable populations. In the post-pandemic future, mental health specialist video consultations in primary care can directly address patients’ preference for holistic management while maintaining therapeutic and spatial separation between mental and physical health [79].

## Supplementary Information

**Additional file 1.** Interview Guide for Patients.

**Additional file 2.** Summary of the Key Themes.

## Data Availability

All data generated or analysed during this study are included in this published article and its supplementary information files.

## Abbreviations

GAD-7: Generalized Anxiety Disorder 7 Scale
PCP: Primary care physician
PHQ-9: Patient Health Questionnaire 9
PROVIDE: ImPROving cross-sectoral collaboration between primary and psychosocial care: An implementation study on VIDEo consultations
PST: Problem-solving therapy
