# Supplementary material for: Primary care patients’ experiences of video consultations for depression and anxiety: a qualitative interview study embedded in a randomized feasibility trial
Source: BMC Health Serv Res. 2023 Jan 4;23:9. doi: 10.1186/s12913-022-09012-z (PMC9811759; doi:10.1186/s12913-022-09012-z)
Supplement: Supplementary file 1 — Additional file 1. Interview Guide for Patients. [file 12913_2022_9012_MOESM1_ESM.pdf]

Additional File 1. Interview Guide for Patients

(Finalized version as of June 2020)

- How were the video consultations delivered?
- What was your experience with the video consultations like?
- To what degree were you satisfied with the video consultations?
- What worked out for you? What did not go well?
- If applicable, which logistical problems did you experience? If applicable, how were they dealt with?
- If applicable, which technical problems did you experience? If applicable, how were they dealt with?
- If applicable, how did you deal with connectivity interruptions or failures?
- How do you rate the usability of videoconferencing platform?
- How do you rate the technical support before, during and after the video consultations?
- How did you perceive the intervention? How do you rate its intensity and length?
- How comfortable did you feel in the room where the video consultations were conducted?
- In summary, how much would you say you were able to engage with the video consultations?
- Overall, how did you experience the communication with and the relationship with the mental health specialist?
- If applicable, how did you experience the video consultations in comparison with your previous experiences with same-room therapy?
- Based on your personal experience, if applicable, which advantages do you see from video consultations?
- Based on your personal experience, if applicable, which disadvantages do you see from video consultations?
- What role did the primary care physician play in the implementation of the video consultations?
- How would you say you have benefited from the video consultations and the intervention? If applicable, what was particularly helpful for you?
- Would you engage in the intervention again and/or recommend it to others? Why? Why not?
- Do you have any suggestions for improving the intervention?
- Is there anything else you would like to add?
