# Supplementary material for: Primary care patients’ experiences of video consultations for depression and anxiety: a qualitative interview study embedded in a randomized feasibility trial
Source: BMC Health Serv Res. 2023 Jan 4;23:9. doi: 10.1186/s12913-022-09012-z (PMC9811759; doi:10.1186/s12913-022-09012-z)
Supplement: Supplementary file 2 — Additional file 2. Summary of the Key Themes. [file 12913_2022_9012_MOESM2_ESM.pdf]

Additional File 2. Summary of the Key Themes

Key theme	Subthemes	Definition	Supporting Quote
**Implementation – How well were the mental health specialist video consultations delivered and received?**	Practical aspects	This code comprises all responses related to the tangible aspects of the intervention and its mode of delivery, e.g., the scheduling of appointments.	*"It was always well organized, and just worked out well." (Participant 01)*
	Patients' experience	This code comprises all responses related to the patients' experience during the receipt of the intervention, e.g., concerning the therapeutic relationship or the room where the intervention was conducted.	*"It was interrupted from time to time and also completely disconnected once." (Participant 12)*
	Perceived disadvantages	This code captures all responses pointing to disadvantages of the intervention and its mode of delivery. *(Do not use this code when the patient refers to external factors that negatively affected the consultation)*	*"Maybe one disadvantage is that she [the mental health specialist] is not directly sitting in front of me." (Participant 13)*
	Suggestions for modifications	This code captures all suggestions for improving the intervention and its mode of delivery.	*"maybe a bit friendlier or a bigger screen" (Participant 16)*
**Mechanism of impact – How did the mental health specialist video consultations work?**	Patients' experience concerning the intervention	This code captures all responses that elaborate on intervention components from which patients benefited. *(Do not use this code when the patient refers to general advantages/disadvantages of video consultation)*	*"[it] actually felt longer, it was just very intense, and the chemistry was right, and I was able to engage fully immediately." (Participant 4)*

	Patients' experience concerning video consultations as the mode of delivery	This code captures all responses that elaborate on aspects related to the mode of delivery from which patients benefited, e.g., familiar environment of the primary care practice.	*"The biggest advantage for me was (...) that I got a quick appointment, that I was helped." (Participant 13)*
	Overall conclusion	This code captures all responses that refer to an overall conclusion on intervention and mode of delivery.	*"I can only recommend it; I would say it [the MHSVC intervention] is a good way." (Participant 01)*
**Context - Which external factors influenced the delivery and functioning of the consultations?**	Facilitators	This code captures all responses context factors facilitating the implementation of the intervention, i.e., when the patient values shorter travel distances as an advantage of the video consultation.	*"One advantage is that you only have to go 'to the doctor', to the primary care physician." (Participant 07)*
	Barriers	This code captures all responses context factors complicating the implementation of the intervention, i.e., when the patient values shorter travel distances as an advantage of the video consultation.	*"You do need the therapists to implement this [the intervention]. I wonder whether you find enough mental health specialists […] I do see that as a disadvantage." (Participant 03)*
